# The Texas Physicians

**Published:** 1898-01

**Authors:** 


THE TEXAS PHYSICIANS.—The JOURNAL is daily in receipt of letters insisting that we urge upon the profession the necessity of concerted action and prompt and vigorous efforts to secure a repeal of the iniquitous tax on practicing physicians. We are pleased to see that in many of the county societies resolutions have been adopted condemning the tax, and urging the appointment of representatives of the profession by the State Medical Association, next meeting, to go to Austin at the next session of the legislature, and petition for the repeal of the obnoxious act. The State Medical Association, at its next meeting, which will take place at Houston, on the fourth Tuesday in next April, should, and doubtless will, take some action; meantime, let every local organization move in the matter. Let the news go forth that the medical profession will not only not support any candidate for the legislature who will not promise in advance to vote for a repeal of the tax, but that they will actively oppose any such candidate. In some counties, medical men have already volunteered to stump the county in the interest of the candidate who will pledge himself to aid us. Let each organization, at the meeting to be held last before the meeting of the State Medical Association, elect delegates to the State Association and send them instructed in this matter. In no other way will we be able to get a hearing. The profession must make its influence at the polls felt; for it seems that they have very little elsewhere. Before a man is elected, he will talk very nicely to get the vote and influence of the doctors; after he is elected for two years, he is quite independent of the doctors. A strong pull and a long pull and a pull altogether, must be made, if we would relieve ourselves of this unjust tax. The town and county, following the example of the State, have in many instances levied a tax also, so that it amounts to $10 instead of $5.

